# Influence of Mast Cells in Drug-Induced Gingival Overgrowth

**DOI:** 10.1155/2013/275172

**Published:** 2013-01-28

**Authors:** Tamilselvan Subramani, Vidhya Rathnavelu, Swee Keong Yeap, Noorjahan Banu Alitheen

**Affiliations:** ^1^Department of Cell and Molecular Biology, Faculty of Biotechnology and Biomolecular Sciences, University Putra Malaysia, 43400 Serdang, Selangor, Malaysia; ^2^Department of Oral and Maxillofacial Pathology, Faculty of Dental Science, Sri Ramachandra University, Porur, Chennai 600116, India; ^3^Institute of Bioscience, University Putra Malaysia, 43400 Serdang, Selangor, Malaysia

## Abstract

Mast cells (MCs) are multifunctional effector cells that were originally thought to be involved in allergic disorders. Now it is known that they contain an array of mediators with a multitude of effects on many other cells. MCs have become a recent concern in drug-induced gingival overgrowth (DIGO), an unwanted outcome of systemic medication. Most of the studies have confirmed the significant presence of inflammation as a prerequisite for the overgrowth to occur. The inflammatory changes within the gingival tissue appear to influence the interaction between the inducing drug and the fibroblast activity. The development of antibodies to MC-specific enzymes, tryptase and chymase, has facilitated the study of mast cells in DIGO. Many immunohistochemical studies involving MCs have been conducted; as a result, DIGO tissues are found to have increased the number of MCs in the gingiva, especially in the area of fibrosis. At the cellular level, gingival fibrogenesis is initiated by several mediators which induce the recruitment of a large number of inflammatory cells, including MCs. The purpose of this paper is to access the roles played by MCs in gingival overgrowth to hypothesize a relationship between these highly specialized cells in the pathogenesis of DIGO.

## 1. Introduction

**Figure 1:**
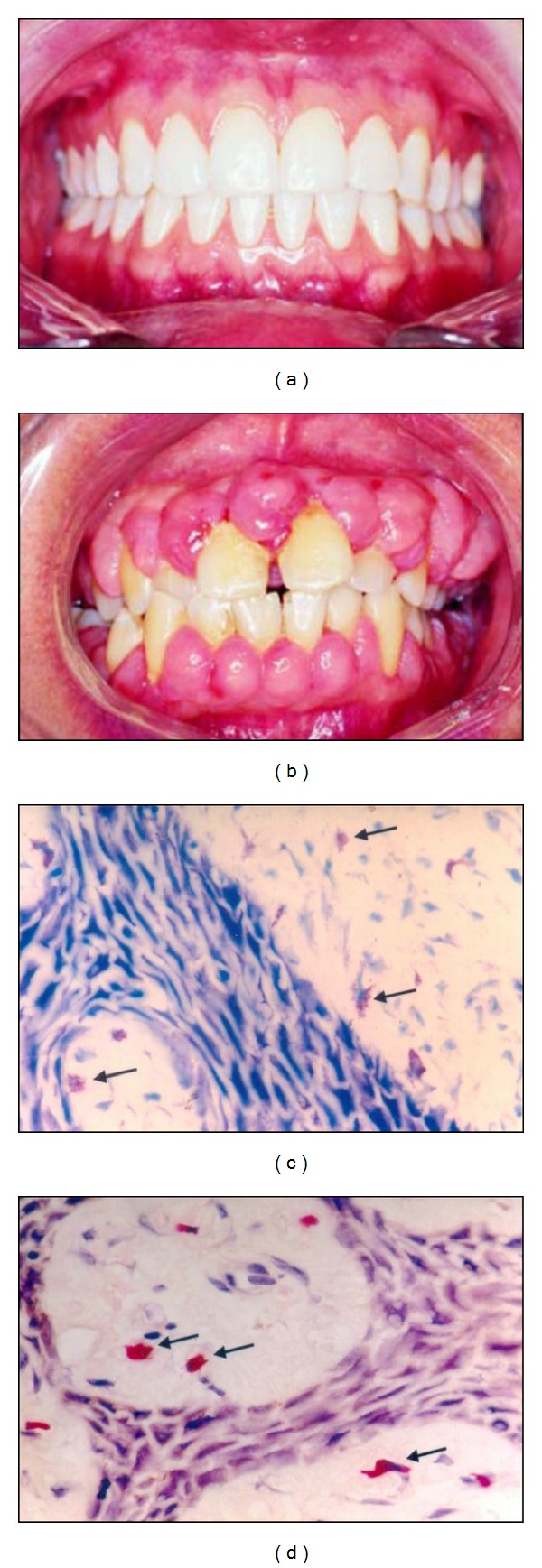


Drug-induced gingival overgrowth (DIGO) is previously termed as gingival hypertrophy or gingival hyperplasia by finding an increased number of fibroblasts in gingival connective tissues [1]. However, these earlier terms did not accurately reflect the histologic composition of enlarged gingiva. It is characterized by massive accumulation of extracellular matrix (ECM) components or decrease in the degradation of ECM of the gingival connective tissue and/or a combination of both of these mechanisms [2]. DIGO is an adverse effect with three types of drugs: phenytoin (Phe), an antiepileptic; cyclosporine-A (CsA), an immunosuppressant; and calcium channel blockers, such as nifedipine (Nif), which are widely prescribed for the treatment of various diseases [3–5]. Drug-induced gingival enlargement consists of soft tissue growth that begins at interdental papilla then develops to papilla and increases to marginal gingiva. As the tissue enlarges it develops a characteristically thickened and lobulated appearance (Figure 1). It may partially or completely cover the tooth surfaces, including the occlusal surfaces, as well as extending the other way, into the sulcus. The epithelial surface is usually smooth and fibrotic, but can be nodular in CsA-induced gingival enlargement [6]. If there is underlying periodontal disease then the tissues may be inflamed, red, or purplish in colour, and highly vascularised, with a tendency to bleed profusely [7]. Gingival enlargement tends to be more severe in areas where plaque accumulates, such as at the edges of fillings and around orthodontic appliances. Though clinically they may look like fibrosis, histopathological and biochemical studies have shown increased cells, collagen, and ECM and hence the term gingival overgrowth was coined [4, 5]. Furthermore, the presence of various degrees of gingival inflammation is one reason for difficulty in the accurate assessment of DIGO, because inflammation acts as an exacerbation factor of gingival overgrowth. At the cellular level, gingival fibrogenesis is initiated by several mediators which induce the recruitment of a large number of inflammatory cells, including mast cells (MCs) [8]. MCs are tissue-specific multifunctional cells, derived from hematopoietic progenitor in bone marrow. They do not develop into mature terminally differentiated mast cells until reaching the tissue or organ in which they become resident. They locate close to blood vessels, epithelia, and nerves in connective tissues, allowing their input in homeostatic functions and also being primary immune barriers [9, 10]. Their anatomic distribution and structural relationships allow mast cells to modulate innate and adaptive immune responses; however, this role requires mast cell activation to stimulate cell degranulation together with release of mast cell mediators. In chronic inflammation, mast cells produce a range of bioactive amines, chemokines, cytokines, proteoglycans, and growth hormones, which mediate a diverse range of mast cell function [11]. Accordingly, this paper will describe the role of mast cells in gingival tissues as well as addressing what is currently known in regard to gingival mast cell and its influence on drug-induced gingival overgrowth.

## 2. Gingival Mast Cells and Fibrosis 

Mast cells are found in all tissues of the oral cavity, including the gingival tissues [12, 13]. It has been reported that mast cells were constantly present in healthy gingival tissue and located between epithelial cells and connective tissues [14, 15]. Proinflammatory cytokines that are released during the initial stage of the inflammation influence the migration of mast cells. In chronic periodontitis, there was an increase in the number of mast cells that may participate either in the destructive events or in the defense mechanisms of periodontal disease by secreting cytokines [16]. Following degranulation, mast cell mediators are deposited in large quantities in the extracellular environment, where they exert effects on endothelial cells and other cell types. The importance of mast cells in a number of pathological processes is beyond doubt, but because of their poorly defined physiological functions and phenotypic heterogeneity, they have always been a controversial issue [11]. It is now thought that mast cell-specific proteases (chymase and tryptase) represent the main criterion of differentiation: mast cells containing only tryptase (MC_T_) can be distinguished from those containing both tryptase and chymase (MC_TC_). Importantly, in several models both MC_T_ and MC_TC_ were present and directly involved in gingival fibrosis [8, 12]. Chronic periodontitis and fibrosis involve a common sequence of events requiring the interaction of endothelial cells with infiltrating mast cell. Early events in this process, profibrotic stimuli, include key growth factors, transforming growth factor-*β* (TGF-*β*) [13], and fibroblast growth factor [14] as well as inflammatory cytokines and chemokines facilitating mast cell recruitment and activation. The release of proteolytic enzymes like matrix metalloproteases (MMP) mediates fibrogenic injury and the overall balance of activators and inhibitors is altered in a manner favoring net matrix deposition. Mast cell effectively supports this process by elaborating the cytokines [15] and chemokines [16]. Mast cell produced mediators such as histamine, heparin, and TNF-*α* which can influence fibroblast proliferation, ECM synthesis, and degradation. TNF-*α* also upregulates C-C chemokine receptor type 1 (CCR1) and regulated, on activation, normal T cell expressed and secreted (RANTES) expression which in turn triggers further mast cell degranulation [17]. Mast cell chymase is also known to stimulate MMP-9 which mediates basement membrane disruption [18]. On the whole, the cyclic activity of inflammatory mediator secretion and mast cell degranulation may result in the chronicity of gingival fibrosis (see Table 1 for details).

## 3. Renin Angiotensin System and Mast Cell

The gingiva provides all of the essential components for a functional renin angiotensin system (RAS), and there is an increasing evidence of the contribution of the local RAS in the pathogenesis of gingival overgrowth [19–21]. Angiotensin II (Ang II) is the effector peptide of RAS, which acts as a major role in regulation of collagen synthesis and growth modulating effects on fibroblasts [22–24]. In its target cells, Ang II binds to different subtypes of G protein-coupled receptors and induces constriction [25], hypertrophy, and proliferation [26] via the angiotensin II type 1 (AT1) receptor. Angiotensin II type 2 (AT2) receptors mediate vasodilatation, growth inhibition [27], and apoptosis [28]. It has been known for some time that there are alternative pathways for converting Ang I to Ang II that do not require angiotensin converting enzyme (ACE). The accumulating evidence demonstrated the alternative Ang II-generating pathway through the enzyme mast cell chymase [29]. Mast cells synthesize renin and release during degranulation that can generate Ang II from Ang I [21, 30]. Nurmenniemi et al. 2004 have demonstrated the augmented mast cell and its enzyme chymase and tryptase in healthy human gingival connective tissue [8]. Despite the great number of studies on mast cell populations, at the present time studies regarding the stimulation of local RAS by mast cells in human gingiva are very few. Mariani et al., 1996, examined the overgrowth that affected gingiva of patients treated with cyclosporine after kidney transplant to evaluate the involvement of connective tissue [6]. The ultrastructural study showed that the dimensional increase was largely due to increased production of amorphous ground substance by fibroblasts, possibly resulting from an increased release of mast cells mediators. Furthermore, chymase inhibitors prevented Ang II and TGF-*β* generation in a model of cardiac and kidney fibrosis [31, 32]. In gingival fibrosis, chymase-positive mast cells infiltrate more in numbers in association with increased expression of stem cell factor and IL-8, known mast cell attractants. This was prevented by treatment with ACE inhibitors, suggesting a feedback link between Ang II-induced mast cell chemoattractants and ACE-independent generation of Ang II by mast cells [33]. Administration of a mast cell stabilizer reduces fibrosis and the number of chymase-positive mast cells, without affecting TGF-*β* expression, consistent with a role for fibrosis-induced by chymase-generated AngII [34].These studies collectively provide evidence for mast cell-induced gingival fibrosis by leading to mast cell activation of local RAS.

## 4. Endothelin and Mast Cell

Among the various mast cell mediators and their roles in gingival overgrowth, the function of endothelin-1 (ET-1) is particularly intriguing. Mast cells have been shown to directly produce this peptide [60]; thus, ET-1 may act in an autocrine mode in its release of mast cell mediators. Moreover, mast cells are likely to contribute indirectly to the formation of endothelin by releasing chymase, which can generate ET-1, by cleavage of big ET-1 [61]. ET-1 is a powerful vasoconstrictor with mitogenic activity on vascular smooth muscle and fibroblasts. There are currently two distinct human endothelin receptors known, ETA and ETB, which are members of the seven transmembrane G protein-coupled rhodopsin superfamily [62, 63]. Activation of both ETA and ETB on smooth muscle cells leads to vasoconstriction, whereas activation of ETB located on endothelial cells leads to vasodilation by increasing NO production [64]. Mounting evidence has strongly correlated elevated circulating and tissue levels of endothelin (ET)-1 in drug-induced gingival overgrowth in patients and animal models [65, 66]. Mast cells store and release several products that are capable of activating MMPs. Although increased MMP activity has been observed in drug-induced gingival fibrosis, the recent findings implicate gingival mast cell degranulation as an *in vivo* mechanism responsible for activating MMPs and the subsequent ECM degradation [39, 40]. Studies linking peritoneal and skin mast cells to tissue remodelling have shown that mast cell tryptase can activate interstitial collagenase (MMP-1) and stromelysin (MMP-3) under *in vitro* conditions [41, 42]. Studies using peritoneal mast cells isolated from mice have shown that chymase can activate MMP-2 and -9 [43]. Moreover, prevention of MMP-2 activation by treatment with the mast cell stabilizer nedocromil sodium firmly establishes that ET-1-induced MMP-2 activation is mediated via degranulation of resident mast cells [44]. In addition, growing evidence from other tissues suggests that ET-1 induces the expression of many types of MMPs as well as ECM protein expression, including collagens, laminin, and fibronectin (FN), in cell culture and animal models [39, 45]. Further, RAS activation is probably the most important mechanistic link between mast cells and gingival fibrosis. In gingival overgrowth, locally formed ANG II will also stimulate ET-1 release and contribute to fibrosis. ET-1 was produced by cultured endothelial cells and MCs after Ang II stimulation [67, 68]. Furthermore, Ang II increases the production of ET-1 by human MCs and subsequently contributes to its mitogenic effects [69]. On the other hand, ET-1 enhanced the conversion of Ang I to Ang II in pulmonary artery endothelial cells [70]. Thus, the role of mast cells in the generation of fibrosis involves the direct effect of mast cell-derived endothelin-1 and angiotensin II, the exacerbation of preexisting inflammatory mediators, and the release of mast cell enzymes, such as renin and chymase.

## 5. Experimental Evidence Exploring the Role of Mast Cells in Gingival Overgrowth 

**Figure 2:**
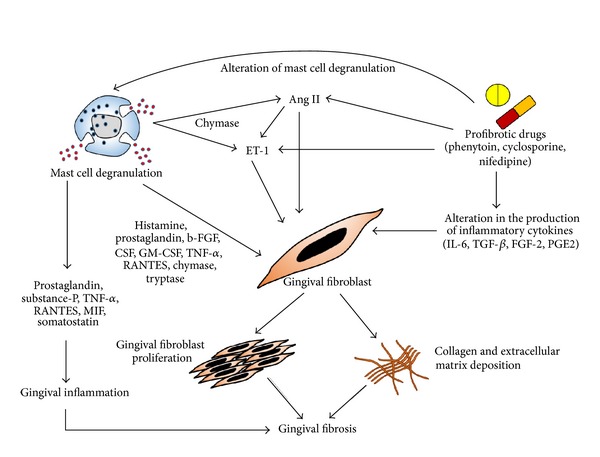


A number of studies have found that mast cells are involved in gingival overgrowth thus showing that they play a broader role than originally thought. It involves a multistage inflammatory process with fibroblast activation, inflammatory cell infiltration, and ECM expansion that is orchestrated by a network of cytokines/chemokines, growth factors, adhesion molecules, and signaling processes (Figure 2). *In vitro* studies have pointed out the potential for mast cells to induce fibroblast proliferation and collagen synthesis. Takeda and colleagues reported the marked elevations in total amount of mast cell granules in gingival fibroblast. This finding was interpreted as phagocytosis of mast cell granules by fibroblasts and suggested that mast cells play some role in fibroblast activity in human connective tissue [71]. The immunohistochemical expression of mast cell tryptase in inflammatory fibrous gingival hyperplasia demonstrated the involvement of mast cells in the induction of fibrosis by observing increased mast cell degranulation in fibrous tissues [48]. MC tryptase, by virtue of its capacity to activate fibroblast-expressed protease-activated receptors eventually in conjunction with heparin, could be an important mediator in this effect. MC chymase could also play a role. Indeed, chymase-positive gingival overgrowth patients showed significantly increased fibrotic lesions in the gingival tissues compared with chymase-negative patients [49]. A recently published study demonstrated that mast cell tryptase and chymase were expressed significantly higher in DIGO tissues compared to healthy gingiva and may contribute to alterations in basement membranes [8]. In one clinical study on CsA induced gingival overgrowth found the dimensional increase of amorphous ground substances by fibroblasts and this was possibly resulting from an increased release of mast cell mediators [6]. In contrast to this, Asahara et al., 2000, demonstrated that mast cells are not necessary in the development of cyclosporin A-induced gingival hyperplasia, and that the increased number of mast cell observed in the enlarged gingiva may be a secondary effect of gingival hyperplasia [72]. On the other hand, it has been shown that mast cells were found to be increased in nifedipine as well as phenytoin-induced gingival overgrowth and mast cell chymase facilitating the augmented expression of TGF-*β* [31]. The chymase also increases cell proliferation and type I collagen synthesis in fibroblasts and chymase inhibitors completely suppress this increased proliferation [73, 74]. One study demonstrated the significant stimulation of 5 alpha-reductase activity in human gingival fibroblasts by mast-cell histamine, alone and in combination with phenytoin [35]. The findings suggested a novel hypothesis that mast-cell-mediated androgen action in the gingiva in response to phenytoin could contribute to gingival overgrowth. On the other hand, an ultrastructural study of the CsA-induced gingival overgrowth tissues showed the increased production of amorphous ground substance by fibroblasts, possibly resulting from an increased release of histamine by mast cells [36]. Mast cells have been associated with fibrosis in other organs besides gingival tissues, including kidney, heart, lung, and liver.Models of gingival fibrosis in mast cell-deficient mice have not been extensively studied. The available *in vitro* data [6, 8, 37] showed the functional role of mast cells in fibrogenesis and suggested the net outcome of mast cell involvement as a profibrotic stimulator.

## 6. Conclusion

In summary, mast cells participate in many inflammatory oral diseases, particularly those associated with fibrosis. They possess very diverse roles ranging from proinflammatory to immunomodulatory. Upon their activation, they promote the local RAS generation consequently able to stimulate endothelin and other profibrotic mediators. What can be observed from the studies being done in this field is that there are some consistent findings as well as some fragmented findings. Taken together, these findings clearly demonstrate the central role of mast cells in DIGO; however, future research will contribute significantly to the understanding of the influence of mast cells in DIGO.

## Figures and Tables

**Table 1:** Mast cell mediators and their roles in gingival tissue.

Mediators	Model	Pathophysiological effect	References
Histamine	Human gingival fibroblast, skin fibroblast	Fibroblast proliferation and collagen synthesis	[17, 35, 36]
Serotonin	Rat gingival tissues	Vasoconstriction	[37]
Arylsulphatases	Human gingival tissue	Lipid and proteoglycan hydrolysis	[38]
MMP 1–3, 9, 10	Human gingival tissues	Matrix degradation	[39–45]
Carboxypeptidase A	Human gingival tissues	Peptide processing	[38]
Kininogenases	Rat gingival tissues	Synthesis of vasodilatory kinins	[46]
Phospholipase	Human gingival fibroblast	Fibroblast proliferation, arachidonic acid generation	[47]
Chymase	Human gingival fibroblast, human gingival tissues	Fibroblast proliferation, angiotensin II generation,	[8, 12, 29]
Tryptase	Human gingival fibroblast, human gingival tissues	fibroblast proliferation, inflammation	[8, 41, 42, 48, 49]
TGF-*β*	Human gingival fibroblast, human gingival tissues	Collagen synthesis, fibronectin generation, reduce MMP-1 synthesis	[13, 31, 32, 34]
b-FGF	Human gingival fibrobalsts, gingival tissues	Fibroblast proliferation	[50]
CSF, GM-CSF	Human gingival fibroblast, human gingival tissues	Fibroblast proliferation, collagen synthesis, cytokine generation	[13, 50, 51]
Chondroitin sulfate, Heparin, Hyaluronic acid	Human gingival fibroblast Human gingival tissues	Inflammation, regulation of cell addition and trafficking	[29, 38, 52]
Chemotactic factors	Human gingival tissues	Leukocytes infliltration	[51]
Corticotropin releasing factor, Endorphins, Somatostatin	Human gingival tissues and crevicular fluid, Rat oral tissues	Vasodilatation, analgesia, inflammation	[38, 53, 54]
Bradykinin	Human gingival tissues, mouse oral tissues	Antiinflammatory	[54]
Substance P	Human gingival fibroblast	Inflammation	[54]
Vasoactive intestinal peptide	Human gingival tissues Rat mandibular gingiva	Vasodilatation	[54]
Leukotriene	Human gingival tissues, human gingival fibroblast, rat oral tissues	Leukocyte chemotaxis	[1, 54–56]
Platelet activating factor	Human gingival tissues, rat tibiae, gingival fibroblasts.	Vasodilatation Vasoconstriction	[3, 53, 54, 56]
Prostaglandin	Human gingival tissues, gingival fibroblast,	Inflammation, fibroblast proliferation	[1, 5, 54, 55]
Nitric Oxide	Crevicular fluid from DIGO developed patients, human gingival tissues	Vasodilatation, neurotransmission	[4, 5, 57]
IL-1, 2, 3, 4, 5, 6, 8, 9, 10, 13, 16.	Human gingival tissues, gingival fibroblast	Inflammation, leukocyte migration, fibroblast proliferation	[13, 38, 50, 54]
INF-*γ*, MIF	Human gingival tissues	Inflammation, leukocyte proliferation, and activation.	[54]
TNF-*α*	Human gingival tissues, gingival crevicular fluid	Gingival inflammation, collagen synthesis, fibroblast proliferation	[29, 38, 50, 58, 59]
RANTES	Human gingival tissues	Chemoattractant, collagen synthesis, fibroblast proliferation, TNF-*α* generation	[17, 54, 55]
MCP-1, 3, 4.	Human gingival tissues	Chemoattractant, gingival inflammation	[54]
